# New Frontiers for Predicting Atrial Fibrillation and Stroke: AI-Based Left Atrial Volumetry

**DOI:** 10.1016/j.jacadv.2024.101299

**Published:** 2024-10-07

**Authors:** Carlotta Onnis, Marly van Assen

**Affiliations:** a Department of Radiology, Azienda Ospedaliero Universitaria (A.O.U.), Cagliari, Italy; b Translational Laboratory for Cardiothoracic Imaging and Artificial Intelligence, Department of Radiology and Imaging Sciences, Emory University, Atlanta, Georgia, USA

**Keywords:** artificial intelligence, atrial fibrillation, cardiac magnetic resonance imaging, coronary artery calcium, left atrial volume, stroke

**Figure:**
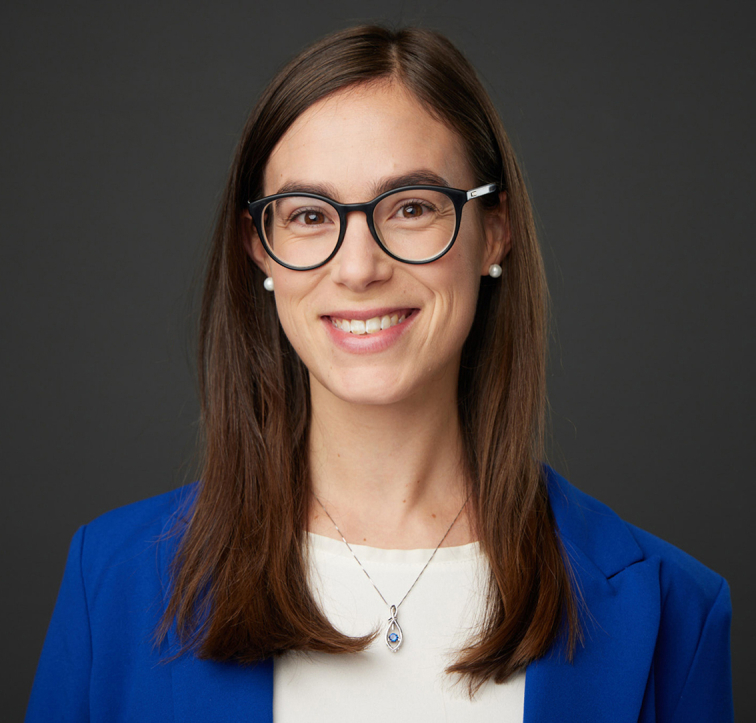

Atrial fibrillation (AF), the most common cardiac arrhythmia, has a global prevalence estimated to be 59 million individuals in 2019.^1^ However, the true prevalence is estimated to be even higher, due to the number of undiagnosed patients, representing 11% to 23% of patients with AF.^2^ AF is a tremendous burden on patients and health care systems not only because of its prevalence and associated costs but also because it increases the risk of stroke 5-fold, a major contributor to cardiovascular mortality.^3^

Timely detection of AF, potentially in asymptomatic patients, could contribute to a decrease time-to-treatment and reduce AF-associated complications. Hence, many risk prediction scores have been developed to evaluate the future risk of occurrence of AF and stroke. For example, the revised Framingham stroke risk score for 10-year stroke prediction and the atherosclerotic cardiovascular disease pooled cohort equation for 10-year prediction of a first atherosclerotic cardiovascular disease event, including stroke.^4^ Other nontraditional risk factors, such as coronary artery calcium (CAC) score, have been assessed for their additive value to these risk scores. With regard to AF, widely used methods to predict risk of new-onset AF include the CHARGE-AF (Cohorts for Heart and Aging Research in Genomic Epidemiology AF) risk score and left atrial size. The former is a 5-year AF risk predictor that uses age, race, medical history, and vital signs as variables, and it has shown significant discrimination for AF incidence with a uniform prediction window.^5^^,^^6^ The latter, particularly atrial volume, has been extensively studied as a marker of increased risk of AF, and left atrial enlargement correlates with increased levels of N-terminal pro b-type natriuretic peptide (NT-pro-BNP).^7^^,^^8^ Traditionally, left atrial volume is measured with cardiac MR (CMR), which can be a costly and time-consuming imaging technique, or with echocardiography, which is a highly operator-dependent modality. Atrial size measurement on computed tomography (CT) scans has been evaluated as a possible alternative. Reference values of left atrial volume have been determined for electrocardiogram-gated contrast-enhanced CT, but studies have also focused on noncoronary measures, including cardiac chambers’ volumetry, quantified on noncontrast electrocardiogram-gated scans, such as CAC scans.^9^^,^^10^ The benefit of using CAC scans is its wide availability and low radiation dose. CAC scans are used as a screening tool for coronary artery disease, and there has been a notable increase in its use in recent years in the United States and worldwide.^11^ Thus, noncoronary measurements, such as left atrial volumetry, may improve its prognostic value beyond prediction of coronary artery disease, and may provide additional information about patients’ cardiac health, without the need for additional tests.

As artificial intelligence (AI) increasingly reshapes medical practice with excellent capabilities for segmentation, new algorithms have been developed and applied for AF detection.^12^ The novelty of the study by Naghavi et al^13^ in this issue of *JACC: Advances* relies on the application of AI in CAC scans to measure left atrial volume, predict future AF and stroke, and overcome the challenges of CMR-based left atrial size quantification.

Firstly, the authors applied an AI-powered tool (AI-CAC) that automatically segments and calculates the volume of cardiac chambers on CAC scans of asymptomatic patients, included in the MESA (Multi-Ethnic Study of Atherosclerosis) study (2000-2002). They compared the resulting AI-based volumetry to human-based volumetry obtain from CMR and showed that their AI tool not only reduced the average time of analysis from 30 to 45 minutes to 21 seconds but also obtained comparable predictive value using CT for AF and stroke to that of CMR. Secondly, the authors compared the predictive value of the AI-based tool with that of the CHARGE-AF score, Agatston score, and NT-pro-BNP for both short- and long-term incidence of AF and stroke. AI-based left atrial volumetry (area under the curve [AUC]: 0.83) significantly outperformed CHARGE-AF (AUC: 0.74), Agatston score (AUC: 0.68), and NT-pro-BNP (AUC: 0.74) for short-term (1-year) AF prediction. AI-CAC (AUC: 0.77-0.80) also outperformed Agatston score (AUC: 0.67-0.69) and NT-pro-BNP (AUC: 0.68-0.71) for 2- to 5-year stroke prediction. For long-term (15-year) prediction of AF and stroke, AI-CAC showed significantly higher AUC (0.80 and 0.76, respectively) when compared to Agatston score (AUC: 0.68 and 0.64) and NT-pro-BNP (AUC: 0.70 and 0.63).

Most importantly, both the CT and CMR-derived left atrial volumes demonstrated a substantial net reclassification index when added to CAC score, CHARGE-AF, and NT-pro-BNP. These results suggest that using left atrial volume as a screening tool among asymptomatic patients may better detect those cases that would be wrongly flagged as low risk when using alternative prediction tools. In this study, the authors present left atrial volume, measured with AI, as a possible reliable, fast tool to reclassify risk for future AF and stroke.

As preventive medicine is increasingly focusing on screening tools for AF, Naghavi et al suggest an original approach to facilitate AF screening in scans that would be performed for other reasons.^1^^,^^13^ They expand the boundaries of the current study beyond CAC scans and suggest the application of AI-CAC volumetry tool to non-gated lung cancer screening CTs as possible future focus of research. The possibility of applying AI-CAC to both CAC scans and lung cancer screening scans is promising, and it would allow screening of a wider population. However, further investigations are warranted to evaluate the predictive value of AI-volumetry for AF and stroke in lung cancer screening CTs. Moreover, the application of AI-CAC to more recently acquired CAC scans using novel scanner technology is also warranted.

In conclusion, this study explores new innovative frontiers for AF and stroke prediction. The opportunity provided by AI-CAC to run in the background and automatically flag cases with an enlarged left atrium, at increased risk of developing AF and stroke, is promising and of great clinical relevance. Further prospective studies are needed to evaluate its clinical applicability and integration into daily workflow.

## Funding support and author disclosures

Dr van Assen has received research funds from Cleerly Inc and Siemens Healthineers. Dr Carlotta has reported that she has no relationships relevant to the contents of this paper to disclose.
